# Association of BMI and WC for insulin resistance and type 2 diabetes among Brazilian adolescents

**DOI:** 10.1016/j.jped.2024.07.007

**Published:** 2024-08-12

**Authors:** Cesar Pirajá Bandeira, Beatriz D. Schaan, Felipe Vogt Cureau

**Affiliations:** a Faculty of Medical Sciences, Graduate Program in Health Sciences: Cardiology and Cardiovascular Sciences, Universidade Federal do Rio Grande do Sul, Porto Alegre, RS, Brazil; b Endocrine Division, Hospital de Clínicas de Porto Alegre, Porto Alegre, RS, Brazil; c Graduate Program in Medical Science: Endocrinology, Universidade Federal do Rio Grande do Sul, Faculty of Medical Sciences, Porto Alegre, RS, Brazil; d Graduate Program in Physical Education, Universidade Federal do Rio Grande do Norte, Natal, RN, Brazil

**Keywords:** Body mass index, Waist circumference, Insulin resistance, Type 2 diabetes mellitus, Adolescents, Obesity

## Abstract

**Objective:**

To investigate how body mass index (BMI) and waist circumference (WC) may be associated with insulin resistance and type 2 diabetes (T2DM) in Brazilian adolescents.

**Methods:**

Cross-sectional study using data from the Brazilian Study of Cardiovascular Risks in Adolescents (ERICA) including adolescents aged 12–17 years. The relationship between adiposity and T2DM was investigated using ordinal logistic regression models. To study the association between adiposity categories and the occurrence of insulin resistance, linear regression models were used.

**Results:**

The prevalence of T2DM for the same BMI category did not increase with the presence of high WC. Regarding insulin resistance, for the same BMI categories, having a high WC resulted in a higher prevalence of insulin resistance (HOMA-IR). The only groups significantly associated with prediabetes and T2DM were those with obesity by BMI with elevated WC (POR 1.68, 95 % CI 1.45; 1.94) and obesity with normal WC (POR 1.58, 95 % CI 1.01; 2.46). Similar findings were observed concerning insulin resistance, where the increased WC had its greatest effect when associated with obesity by BMI (β Coefficient 2.20, 95 % CI 1.89; 2.50).

**Conclusion:**

The combination of BMI and WC is better for assessing adolescents at risk of developing T2DM.

## Introduction

Obesity is a chronic and complex disease characterized by excess adiposity that affects an individual's overall health. The increase in obesity prevalence is observed in adults, children and adolescents. Obesity is a fundamental pathophysiological factor in the development of cardiovascular risk factors, including type 2 diabetes mellitus (T2DM).^1^ Most patients with T2DM have insulin resistance and insulin secretion dysfunction, where the increased demand for insulin action caused by resistance is not compensated by insulin secretion.^2^ The Homeostasis Model Assessment for Insulin Resistance (HOMA-IR) is widely used to assess insulin resistance and has been shown to be valid in adolescents compared to the gold standard method.^3^ Glycated hemoglobin (HbA1c) is the standard test for assessing glucose control in diabetes and also for diagnostic purposes.^4^

Data from the third National Health and Nutrition Examination Survey (NHANES III) demonstrated that the prevalence of diabetes remains high, making it one of the most common non-communicable diseases with a significant impact on the population's health.^5^ The increase in prevalence has also occurred in the pediatric population: recent data from the United States suggest an incidence of approximately 5000 new cases of type 2 diabetes per year in adolescents.^4^ In Brazil, recent studies suggest that 3.3 % of adolescents aged 12–17 years have type 2 diabetes.^6^ In addition, according to another study based on the same database, 1/5 of adolescents in Brazil have elevated levels of glycated hemoglobin (Hb1Ac).^7^ Thus, obesity can lead to an increased prevalence of cardiovascular risk factors in children and adolescents, which tend to persist into adulthood and continuously accelerate the process of vascular damage.^6^

In this context, exploring anthropometric indices that correlate with obesity, via inexpensive, practical, and safe tools for cardiovascular risk assessment, can greatly contribute to the prevention and early treatment of obesity and T2DM.^8^ The main accepted diagnostic tool and frequently used predictor of morbidity and mortality for various chronic diseases, including T2DM, is body mass index (BMI). However, limitations of BMI, such as the lack of accuracy in assessing adiposity and the fact that it does not reflect the regional distribution of body fat, are questioned.^9^ A systematic review with meta-analysis showed that paradoxically, those individuals who were overweight had lower all-cause mortality than the normal BMI group.^10^ Thus, individuals within the same BMI range can have distinct metabolic characteristics, which can be partially attributed to the presence of central obesity and abdominal fat deposition.^11^ In this regard, other anthropometric measures, such as waist circumference (WC), have gained importance in clinical research since it has been suggested as a more accurate indicator of visceral fat than BMI in adults.^8^ However, this relationship has not yet been fully explored in children and adolescents. Recent research has shown that children who had increased BMI at age 15 were more likely to have an increased risk of insulin resistance and waist circumference at age 16. Therefore, incorporating WC measurements alongside BMI could be a valuable tool for screening cardiovascular risk factors, such as T2DM in adolescents, especially in those with a normal BMI.^12^^,^^13^

This study aims to investigate the association between the combinations of BMI and WC with insulin resistance and T2DM, in a representative sample of Brazilian adolescents.

## Subjects, materials, and methods

### Study design and sample

This study used data from the Study of Cardiovascular Risks in Adolescents (ERICA), which was held between 2013 and 2014. ERICA is a national, cross-sectional, school-based study that aimed to estimate the prevalence of cardiovascular risk factors that are part of metabolic syndrome in a representative sample of Brazilian adolescents aged 12–17 years.

Adolescents attending the seventh, eighth, and ninth grades of primary education and the first, second, and third years of secondary education in public or private schools, in Brazilian capitals and municipalities with more than 100,000 inhabitants, were selected. The choice of these grade levels aimed to include as many adolescents aged 12–17 as possible since there was no information based on individual data for sample selection. All students in the selected classes were invited to participate. A more detailed description of the study design and sampling procedure is available in other publications.^14^

### Participants

For this analysis, data from students who underwent fasting blood collection for 10–12 h (those attending morning classes) were used. The data were collected from 124 municipalities, including 925 public and private schools. Approximately 52 % of the total sample of adolescents had all proposed measurements collected, including blood collection. These measurements include demographic and anthropometric variables, blood pressure measurements, fasting glucose, insulin and glycated hemoglobin (HbA1c).^14^ Adolescents who were pregnant, had type 1 diabetes, and those with physical or mental disabilities that prevented their participation in the study were excluded from the analyses. A complete description of the response rate of ERICA is available in another publication.^15^

All adolescents who agreed to participate in the blood collection provided written informed consent, and informed consent signed by parents or legal guardians. ERICA was approved by the Research Ethics Committees of all 27 Brazilian Federative Units. Specific approval from the Ethics Committee of the Hospital de Clínicas de Porto Alegre was also obtained (protocol number 2009-0098).

### Data collection

#### Assessment of socioeconomic, demographic, and psychosocial data

Data regarding macroregions of origin (North, Northeast, Midwest, Southeast, and South), gender, age, self-reported skin color, type of school (public or private), socioeconomic status (SES), and psychosocial information, such as smoking, alcohol consumption, and weekly physical activity duration (in minutes) were obtained through a self-administered questionnaire using an electronic data collector completed by the adolescents.

To determine the weekly amount of time spent in physical activity, the authors multiplied the self-reported duration and frequency for each listed activity and then dichotomized it into < 420 or ≥ 420 min/week.

#### Assessment of end points

Prior to blood collection, participants were instructed to fast for 12 h overnight, and all blood samples were analyzed in a single laboratory following a standardized protocol. For the outcome assessment, fasting blood glucose, fasting insulin levels, and HbA1c were measured. Additionally, previous medical diagnoses and medication use for diabetes were evaluated through a questionnaire. The diagnostic criteria of the American Diabetes Association (ADA) were employed to define prediabetes and diabetes.^4^ Prediabetes was defined as fasting glucose levels between 100 and 125 mg/dL (5.6–6.9 mmol/L) or HbA1c between 5.7 % and 6.4 % (39–47 mmol/mol). New cases of T2DM were identified by fasting glucose levels ≥126 mg/dL or HbA1c ≥6.5 %. Participants who reported insulin use were classified as having type 1 diabetes mellitus (*n* = 33) and were excluded from subsequent analyses in this study.

To calculate the homeostatic model assessment of insulin resistance (HOMA-IR), the formula: fasting insulin × fasting glucose/22.5 was used. The cutoff points used to indicate insulin resistance were 2.32 for girls and 2.87 for boys. These cutoff points were determined based on the largest areas under the receiver operating characteristic (ROC) curves when analyzing the association of HOMA-IR with metabolic syndrome in Brazilian adolescents.^16^

#### Assessment of anthropometric data

The following anthropometric variables were measured by trained researchers: waist circumference (WC), weight, and height. These measurements were monitored throughout the data collection process using quality control procedures to ensure accuracy and consistency.

Waist circumference was measured using an inelastic tape measure of the brand Sanny, with a precision of 0.1 cm, at the midpoint between the lower curvature of the last fixed rib and the upper curvature of the iliac crest, with the adolescent standing, arms at the sides, feet together, and relaxed abdomen. Abdominal obesity was defined according to cutoff points recommended by the International Diabetes Federation based on the metabolic syndrome criteria for this age group (under 16 years: ≥ 90th percentile for sex and specific age; 16 years and older: ≥ 90 cm for boys and ≥ 80 cm for girls).^17^

Weight was measured using an electronic scale of the brand Líder (model P200M) with a capacity of up to 200 kg and an accuracy of 50 g. Height was measured twice using an Altura Exata portable stadiometer, with an accuracy of 0.1 cm, and the average of the two values was used. Weight and height measurements were used to calculate body mass index (BMI) (weight/height²) for the classification of nutritional status. The BMI-for-age and sex curves proposed by the World Health Organization (2007) were used for the classification. Adolescents were considered to have adequate nutritional status if their z-score was > −2 and ≤ +1, overweight if their z-score was > +1 and ≤ +2, and obese if their z-score was > +2.^18^

For analysis purposes, participants were classified into the following groups based on the combinations of their anthropometric measurements: 1. Normal BMI with normal WC; 2. Normal BMI with increased WC; 3. Overweight BMI with normal WC; 4. Overweight BMI with increased WC; 5. Obese BMI with normal WC; 6. Obese BMI with increased WC.

### Statistical analysis

To obtain representative results for the studied population, all analyses were conducted using sample weights calculated for ERICA, considering the complex survey design. Continuous variables followed a normal distribution and were described by their mean and 95 % confidence intervals (95 % CI). Categorical variables were presented as proportions, followed by their respective 95 % CI.

The relationship between adiposity and prediabetes or diabetes was investigated using ordinal logistic regression models. The proportional odds ratio (POR) estimates the cumulative odds of being in a higher category compared to all lower categories, assuming that the distance between each category is equivalent. The parallel lines assumption, assessed by the Brant's test, was not violated. The Wald's test was used to investigate the linear trend between exposure categories and the outcomes of interest. Furthermore, the authors provide an estimation of Cohen's d based on the ORs, as suggested by Chen et al., to quantify the effect size.^19^

To study the association between BMI and WC groups with HOMA-IR values, linear regression models were employed, and the results were expressed as β coefficient and 95 % CI. Unweighted eta-squared and their 95 %CI were calculated to estimate the effect size in this association.

Both models were adjusted for region, sex, age, self-reported skin color, type of school, and socioeconomic level. The variables included in the model were selected based on the literature and were retained in the model after inclusion. The overall fit of the model was evaluated, and no evidence of multicollinearity was found in the models.

The authors calculated the multiplicative interaction between BMI and WC for the outcomes analyzed. Significant interaction terms were observed for prediabetes and type 2 diabetes (*p* = 0.038) as well and for HOMA-IR (*p* < 0.001).

All tests were two-tailed, and the significance level was set at 5 %. The analyses were performed using Stata version 14 (StataCorp, College Station, TX, USA).

## Results

Table 1 demonstrates the main characteristics of the study population. Among the participants, over half were female, with a mean age of 14.6 (± 14.6; 14.7) years. The Northeast region had the highest number of participants. Most participants reported having mixed skin color and attended public schools. In terms of the total number of participants, 73.3 % were classified with a normal BMI, 17.5 % and 9.2 % with overweight and obesity, respectively. Regarding WC, 12.6 % had an increased WC. The prevalence of T2DM was 3.3 %, with most of the adolescents already having a prior diagnosis before the data collection (90 %, 1136 out of 1250), and 25 % of adolescents had insulin resistance.

**Table 1:** Demographic and clinical characteristics of participants. ERICA 2013–2014.

Characteristics	Total sample (n = 37,892)	
	% or mean	(95 % CI)
Sex assigned at birth		
Female	60.0	(59.5; 60.5)
Male	40.0	(39.5; 40.5)
Age (years)	14.6	(14.6; 14.7)
Region		
North	19.3	(18.9; 19.7)
Northeast	31.1	(30.6; 31.5)
Southeast	22.7	(22.3; 23.1)
South	12.5	(12.1; 12.8)
Midwest	14.5	(14.1; 14.9)
Skin color		
White	40.5	(38.6; 42.4)
Black	7.8	(7.0; 8.7)
Mixed (brown)	49.1	(47.3; 50.9)
Yellow	2.6	(2.2; 3.0)
Type of school		
Public	77.8	(72.5; 82.3)
Private	22.2	(17.7; 27.5)
SES (quintile)		
1°	22.9	(21.4; 24.6)
2°	19.9	(18.5; 21.4)
3°	20.5	(19.4; 21.6)
4°	18.5	(17.4; 19.8)
5°	18.1	(16.3; 20.1)
Smoking		
Yes	2.0	(1.7; 2.3)
No	98.0	(97.7; 98.3)
Alcohol consumption		
No	70.2	(68.6; 71.8)
Yes	29.8	(28.2; 31.4)
Physical activity (min/wk)		
< 420	62.7	(61.6; 63.7)
≥ 420	37.3	(36.3; 38.4)
Weight (kg)	57.6	(57.2; 58.1)
Height (cm)	163.5	(163.3; 163.7)
BMI (kg/m²)	21.4	(21.3; 21.5)
BMI categories		
Normal weight	73.3	(71.8; 74.7)
Overweight	17.5	(16.4; 18.7)
Obesity	9.2	(8.4; 10.0)
WC (cm)	72.2	(71.8; 72.6)
WC categories		
Normal	87.4	(86.3; 88.4)
Increased	12.6	(11.6; 13.7)
Fasting glucose (mg/dl)	86.4	(85.9; 86.8)
HbA1c (%)	5.3	(5.3; 5.40)
HOMA-IR (mean)	2.0	(1.9; 2.11)
High HOMA-IR	25.0	(23.4; 26.6)
Previous diagnosis of T2DM	3.0	(2.7; 3.4)
Prevalence of prediabetes and T2DM		
Healthy	74.8	(73.2; 76.2)
Prediabetes	22.0	(20.6; 23.4)
T2DM	3.3	(2.9; 3.7)

SES, socioeconomic status; BMI, body mass index; WC, waist circumference; HbA1c, glycated hemoglobin; HOMA-IR, homeostasis model assessment for insulin resistance; T2DM, type 2 diabetes mellitus.

Table 2 shows the prevalence of prediabetes, T2DM, and elevated HOMA-IR according to combinations of BMI and WC. There was no difference in T2DM prevalence between the groups. However, adolescents with obesity showed a higher prevalence of prediabetes, regardless of WC categories. Regarding insulin resistance, the group with the highest prevalence was the obesity and increased WC group (69.9 %; 95 %CI: 65.4; 74.1).

**Table 2:** Prevalence of prediabetes, T2DM, and high HOMA-IR according to BMI-WC categories. ERICA 2013–2014.

BMI/WC Categories	Total, n (%)	Prevalence, % (95 % CI)									
		Healthy		Prediabetes		T2DM		Normal HOMA-IR		High HOMA-IR	
Total	37,794 (100)	74.8	(73.2; 76.2)	22.0	(20.6; 23.4)	3.3	(2.9; 3.7)	75.0	(74.7; 76.0)	25.0	(23.4; 26.6)
Normal BMI & normal WC	27,843 (72.6)	75.8	(74.3; 77.2)	21.3	(20.0; 22.6)	3.0	(2.6; 3.4)	83.0	(81.6; 84.3)	17.0	(15.7; 18.4)
Normal BMI & increased WC	225 (0.7)	80.1	(64.9; 89.8)	18.2	(9.1; 32.9)	1.7	(0.7; 4.4)	64.5	(41.0; 82.7)	35.5	(17.3; 59.0)
BMI overweight & normal WC	5025 (13.3)	75.0	(71.7; 78.0)	21.1	(18.2; 24.4)	3.9	(2.6; 5.8)	68.5	(65.2; 71.5)	31.5	(28.5; 34.8)
BMI overweight & increased WC	1611 (4.3)	76.6	(72.0; 80.6)	19.1	(15.7; 23.1)	4.3	(2.5; 7.3)	46.9	(40.8; 53.1)	53.1	(46.9; 59.2)
BMI obesity & normal WC	530 (1.5)	61.2	(50.9; 70.5)	33.7	(25.1; 43.5)	5.1	(2.8; 9.3)	62.6	(52.8; 71.4)	37.4	(28.6; 47.2)
BMI obesity & increased WC	2560 (7.7)	65.9	(61.3; 70.1)	29.9	(25.9; 34.3)	4.2	(3.2; 5.4)	30.1	(25.9; 34.6)	69.9	(65.4; 74.1)

BMI, body mass index; WC, waist circumference; HOMA-IR, homeostasis model assessment for insulin resistance; T2DM, type 2 diabetes mellitus.

Table 3 shows the association of combinations of BMI and WC with prediabetes and T2DM. When evaluating the results, the authors observed that the only groups with a significant association with prediabetes and T2DM were those with obesity defined by BMI – obesity with normal WC and obesity with elevated WC. The results remained similar after adjusting for covariates (region, sex, age, self-reported skin color, type of school, and socioeconomic level). Coefficients for the covariates included in the adjusted model are shown in Supplementary Table 1.

**Table 3:** Association of BMI-WC categories with ordinal outcomes of prediabetes and T2DM. ERICA 2013–2014.

	POR (95 % CI)		*p*-value
Crude Association			
Normal BMI & normal WC	Reference		<0.001
Normal BMI & increased WC	0.77	(0.35; 1.69)	
BMI overweight & normal WC	1.05	(0.89; 1.24)	
BMI overweight & increased WC	0.97	(0.76; 1.25)	
BMI obesity & normal WC	1.96	(1.31; 2.95)	
BMI obesity & increased WC	1.61	(1.37; 1.88)	
Adjusted model^a^			
Normal BMI with normal WC	Reference		<0.001
Normal BMI with increased WC	0.97	(0.37; 2.56)	
BMI overweight with normal WC	0.98	(0.83; 1.16)	
BMI overweight with increased WC	1.22	(0.94; 1.58)	
BMI obesity with normal WC	1.58	(1.01; 2.46)	
BMI obesity with increased WC	1.68	(1.45; 1.94)	

BMI, body mass index; WC, waist circumference; T2DM, type 2 diabetes mellitus; POR, proportional odds ratio.

Ordinal logistic regression was used to test the associations.

a Adjusted by region, sex, age, skin color, school type, and socioeconomic status. The equivalent Cohen's d for significant OR in the adjusted model is approximately 0.2, which represents a small effect size.

Table 4 shows the association between combinations of BMI and WC with HOMA-IR as a continuous variable. Except for the group with normal BMI and increased WC, all other combinations showed a significant association compared to the reference group, with the highest association observed in the adolescents with obesity and with increased WC, even after adjusting for covariates. The coefficients for the covariates included in the adjusted model are presented in Supplementary Table 2.

**Table 4:** Association of BMI-WC categories with HOMA-IR as a continuous variable. ERICA 2013–2014.

	β Coefficient (95 % CI)		*p*-value
Crude association			
Normal BMI & normal WC	Reference		<0.001
Normal BMI & increased WC	0.39	(−0.19; 0.99)	
BMI overweight & normal WC	0.57	(0.49; 0.65)	
BMI overweight & increased WC	1.00	(0.85; 1.15)	
BMI obesity & normal WC	0.98	(0.73; 1.23)	
BMI obesity & increased WC	2.19	(1.90; 2.48)	
Adjusted Model^a^			
Normal BMI & normal WC	Reference		<0.001
Normal BMI & increased WC	0.40	(−0.06; 0.87)	
BMI overweight & normal WC	0.58	(0.50; 0.66)	
BMI overweight & increased WC	0.94	(0.80; 1.08)	
BMI obesity & normal WC	0.96	(0.71; 1.21)	
BMI obesity & increased WC	2.20	(1.89; 2.50)	

BMI, body mass index; WC, waist circumference; HOMA-IR, homeostasis model assessment for insulin resistance.

Linear regression was used to test the associations.

a Adjusted by region, sex, age, skin color, school type, and socioeconomic status. Unweighted Eta-squared and 95%CI for BMI-WC term: crude = 0.120 (0.114–0.126); adjusted =0.117 (0.111–0.123).

## Discussion

This study demonstrates that in a representative sample of Brazilian adolescents, increased WC without being combined with obesity diagnosed by high BMI is not associated with the likelihood of an adolescent having prediabetes or T2DM. However, WC provided additional information on BMI, particularly in the obesity range, ensuring greater accuracy in assessing obesity as a risk factor. Furthermore, when analyzing the relationship of the same anthropometric measures with insulin resistance assessed by HOMA-IR, a similar pattern was observed.

Different findings have been described in the literature regarding the relationship between WC and cardiovascular risk factors. In adults, WC is a reliable marker of abdominal obesity as demonstrated by direct measurement methods such as magnetic resonance imaging.^20^ Abdominal fat is considered a complex and highly active endocrine organ, which, through the release of hormones and cytokines, plays an important role in the inflammatory and metabolic processes. Thus, visceral obesity is usually considered a risk factor for T2DM, metabolic syndrome, and atherosclerosis, being associated with overall and cardiovascular mortality, independent of BMI. Among children and adolescents there is divergence on the most accurate anthropometric measure to determine central obesity and its association with cardiovascular risk factors^3^ One study in Iranian adolescents (CASPIAN III) reported that the association between combinations of overall and abdominal obesity differs depending on the evaluated outcome or risk factor.^21^ Pazin et al., demonstrated that increased WC is associated with high blood pressure in children with normal BMI.^22^ Another study was conducted using data from The Bogalusa Heart Study, including children aged 4–18 years, also found an association between abdominal obesity, assessed according to waist-to-hip ratio, and some cardiovascular risk factors (increased LDL cholesterol and triglycerides, decreased HDL cholesterol, and higher prevalence of metabolic syndrome). Among participants with central obesity but normal weight, 5.88 % had metabolic syndrome, while only 0.26 % of those with normal weight but without central obesity had metabolic syndrome.^23^ This was also observed among Chinese children and adolescents, where the presence of abdominal obesity was more strongly associated with metabolic syndrome than BMI.^24^

The present results, consistent with other studies, demonstrate that increased WC is related to HOMA-IR when overweight or obesity is present according to BMI.^3^ These findings support the pathophysiological role of abdominal fat in determining outcomes related to higher cardiovascular risk (collectively recognized as metabolic syndrome). However, the results presented here demonstrate that having only an increased WC, without this alteration being combined with high BMI, does not increase the likelihood of an adolescent having prediabetes or T2DM. One explanation for this result is the latency period between the onset of obesity and the development of T2DM. Obesity and increased WC are determinants of insulin resistance, but due to the disease's pathogenesis involving compensatory beta-cell function and subsequent increased insulin secretion, the time until beta-cell failure and the consequent development of T2DM may be delayed. As hypothesized by Lee, the increase in obesity during adolescence probably leads to a higher prevalence of T2DM in young adults.^25^

Another point to consider is the pathophysiology of youth T2DM. Although it shares characteristics with T2DM in adults, some unique features have been identified. As demonstrated by studies such as TODAY (The Treatment Options for Type 2 Diabetes in Adolescents and Youth) and RISE (Restoring Insulin Secretion), there is a relatively rapid deterioration of beta-cell function in adolescents compared to the slower decline described in adults with T2DM. In these studies, deficient beta-cell function, rather than insulin sensitivity, was a determining factor for loss of glycemic control.^26^ As puberty is associated with physiological changes and a consequent increase in insulin resistance, regardless of changes in body composition, the demand on the pancreas may be higher during adolescence.^27^ Alternatively, a higher degree of obesity and insulin resistance may be required to develop T2DM in this age group, when compared to adults, where the pancreas may be less healthy.^27^ Although the authors did not stratify the present sample by pubertal status, another study using the same database reported that few adolescents (only 0.5 % of the sample) self-classified themselves as prepubertal (Tanner stage 1), so it is possible to infer that most of this sample were in puberty.^7^

The use of WC may be limited due to changes in body composition caused by growth and pronounced development during this period of life. When comparing anthropometric measures and assessment of abdominal fat using imaging methods in adolescents, WC showed a better correlation with visceral adiposity than other anthropometric parameters.^20^ On the other hand, in Mexican adolescents with obesity, the waist-to-hip ratio was superior to WC in identifying cardiovascular risk factors.^28^ The same was suggested by Robert Ross and colleagues, stating that in children and adolescents in the growth phase, the waist-to-height ratio may be more useful for classifying abdominal obesity than just WC.^29^ Nevertheless, although measuring WC is relatively simple, limitations such as the absence of a consensus on the best method of measurement and the existence of different cutoff points varying by sex, age, and ethnicity undermine the utility of this measure.^29^

The present study has some limitations. The relatively small number of individuals in the normal BMI/increased WC and obesity BMI/normal WC categories (0.7 % and 1.5 % of the sample, respectively) may have led to inaccuracies in the analysis. Another point to consider is the possibility of different types of diabetes, such as Maturity-Onset Diabetes of the Young (MODY) and Type 1 Diabetes (T1DM), being present in the sample. Although the study controlled for this bias by excluding patients using insulin, it is possible that some participants without prior knowledge of the disease and diagnosed through laboratory measurements are individuals with type 1 diabetes. Distinguishing MODY from T2DM can be difficult and often requires additional information and tests that are not typically included in large-scale population studies. However, its prevalence is low (2–5 % of diabetes), and, therefore, likely did not interfere with the results.^30^ The main strength of the present study lies in the large, nationally representative multiethnic sample of adolescents in a developing country. Additionally, data collection, anthropometric measurements, and biochemical analyses were standardized and followed specific protocols.

In conclusion, increased WC without association with obesity diagnosed by high BMI is not related to the likelihood of an adolescent having insulin resistance or T2DM. However, when used in conjunction with BMI, particularly in the overweight and obesity range, increased WC ensures greater accuracy in the associations found. Therefore, the combination of BMI and WC is better for evaluating adolescents at higher risk of developing T2DM and should be used together in clinical practice.

## Financial support

Brazilian Department of Science and Technology at the Secretariat of Science and Technology and Strategic Inputs of the Ministry of Health and Funding Authority for Studies and Projects (FINEP) (grant: 01090421) and by the Brazilian National Counsel of Technological and Scientific Development (CNPq) (grants: 565037/2010-2, 405009/2012-7 and 457050/2013-6) and hospital research incentive fund for Clinics in Porto Alegre (FIPE – HCPA) (grant: 09.098) and Brazilian Federal Agency for Support and Evaluation of Graduate Education (CAPES AUXPE/CAPES n. 2426/2023).

## Conflicts of interest

The authors declare no conflicts of interest.

## Appendix Supplementary materials

Image, application 1

## Footnotes

Supplementary material associated with this article can be found in the online version at doi:10.1016/j.jped.2024.07.007.
