# Supplementary material for: Association of BMI and WC for insulin resistance and type 2 diabetes among Brazilian adolescents
Source: J Pediatr (Rio J). 2024 Aug 12;101(1):30–7. doi: 10.1016/j.jped.2024.07.007 (PMC11763841; doi:10.1016/j.jped.2024.07.007)
Supplement: Supplementary file 1 [file mmc1.docx]

**JPED-D-24-00054 - Supplementary Material**

| Supplementary Table 1. Full model estimates for the association of BMI-WC categories with ordinal outcomes of prediabetes and T2DM. ERICA 2013-2014. | | | |
| --- | --- | --- | --- |
|  | **POR (95% CI)** | | **p-value** |
| Region |  | |  |
| North | Reference | |  |
| Northeast | 1.00 | (0.86; 1.17) | 0.989 |
| Southeast | 1.00 | (0.84; 1.18) | 0.971 |
| South | 0.90 | (0.72; 1.12) | 0.358 |
| Midwest | 0.93 | (0.81; 1.08) | 0.358 |
| Sex |  |  |  |
| Female | Reference | |  |
| Male | 1.56 | (1.38; 1.77) | <0.001 |
| Age, years | 0.88 | (0.85; 0.92) | <0.001 |
| Skin color |  |  |  |
| White | Reference | |  |
| Black | 1.53 | (1.23; 1.91) | <0.001 |
| Mixed (brown) | 1.28 | (1.12; 1.47) | <0.001 |
| Yellow | 1.23 | (0.94; 1.61) | 0.134 |
| Type of school |  |  |  |
| Public | Reference | |  |
| Private | 0.71 | (0.60; 0.83) | <0.001 |
| SES, points | 1.03 | (0.98; 1.08) | 0.280 |
| Normal BMI with normal WC | Reference | |  |
| Normal BMI with increased WC | 0.97 | (0.37; 2.56) | 0.955 |
| BMI overweight with normal WC | 0.98 | (0.83; 1.16) | 0.845 |
| BMI overweight with increased WC | 1.22 | (0.94; 1.58) | 0.136 |
| BMI obesity with normal WC | 1.58 | (1.01; 2.46) | 0.044 |
| BMI obesity with increased WC | 1.68 | (1.45; 1.94) | <0.001 |
| BMI, body mass index; WC, waist circumference; T2DM, type 2 diabetes mellitus; POR, proportional odds ratio; SES, socioeconomic status  Ordinal logistic regression was used to test the associations. | | | |

| Supplementary Table 2. Full model estimates for the association of BMI-WC categories with HOMA-IR as a continuous variable. ERICA 2013-2014. | | | |
| --- | --- | --- | --- |
|  | **β Coefficient (95% CI)** | | **p-value** |
| Region |  | |  |
| North | Reference | |  |
| Northeast | 0.051 | (-0.073; 0.175) | 0.424 |
| Southeast | 0.168 | (0.045; 0.292) | 0.008 |
| South | 0.431 | (0.283; 0.579) | <0.001 |
| Midwest | -0.094 | (-0.208; 0.019) | 0.102 |
| Sex |  | |  |
| Female | Reference | |  |
| Male | -0.310 | (-0.402; -0.218) | <0.001 |
| Age, years | -0.071 | (-0.094; -0.048) | <0.001 |
| Skin color |  |  |  |
| White | Reference | |  |
| Black | 0.341 | (-0.076; 0.758) | 0.109 |
| Mixed (brown) | 0.056 | (-0.008; 0.119) | 0.086 |
| Yellow | 0.087 | (-0.177; 0.352) | 0.516 |
| Type of school |  |  |  |
| Public | Reference | |  |
| Private | -0.082 | (-0.121; -0.042) | <0.001 |
| SES, points | 0.005 | (-0.007; 0.017) | 0.452 |
| Normal BMI & normal WC | Reference | |  |
| Normal BMI & increased WC | 0.405 | (-0.060; 0.872) | 0.088 |
| BMI overweight & normal WC | 0.582 | (0.500; 0.663) | <0.001 |
| BMI overweight & increased WC | 0.942 | (0.800; 1.083) | <0.001 |
| BMI obesity & normal WC | 0.964 | (0.715; 1.213) | <0.001 |
| BMI obesity & increased WC | 2.200 | (1.899; 2.501) | <0.001 |
| BMI, body mass index; WC, waist circumference; HOMA-IR, homeostasis model assessment for insulin resistance; SES, socioeconomic status  Linear regression was used to test the associations. | | | |
